# Techniques for randomization and allocation for clinical trials

**DOI:** 10.1590/1677-5449.202400462

**Published:** 2025-01-13

**Authors:** Anna Carolina Miola, Ana Cláudia Cavalcante Espósito, Hélio Amante Miot

**Affiliations:** 1 Universidade Estadual Paulista – UNESP, Faculdade de Medicina de Botucatu, Botucatu, SP, Brasil.; 2 Universidade do Oeste Paulista – Unoeste, Presidente Prudente, SP, Brasil.

**Keywords:** Randomization, Allocation Concealment, Intervention Studies, Bias Reduction, Methodological Validity

## Abstract

Intervention studies require all participants to originate from the same population, with random allocation to intervention groups to ensure comparability. Randomization is crucial for minimizing confounding factors, allowing differences in outcomes to be attributed to the intervention. Simple randomization performs well for large samples (>100 per group), but smaller samples may require block or stratified randomization to balance group sizes and covariates. When randomization isn't feasible, quasi-randomized methods (e.g., based on dates or enrollment order) can help but must compensate with multivariate adjustments. Moreover, blinding and allocation concealment enhance internal validity and reproducibility. Allocation concealment (e.g., sealed envelopes) prevents bias during participant assignment while blinding mitigates detection and performance biases. Precise methodological descriptions in clinical trial registrations and publications enhance study reliability and reproducibility, highlighting the importance of rigorous planning and transparent reporting in intervention research. This article reviews the key concepts of randomization, blinding, and allocation concealment in interventional studies

Quantitative analysis of data collected in intervention studies requires that the sample is drawn from the same population (at each center) and that participants are allocated to groups at random, i.e. with no interference by researchers or participants. This ensures that each individual has the same chance of being designated to any of the intervention groups (IGs).^1-4^

The importance of randomized allocation lies in its capacity to homogenize unknown or unmeasured confounding factors, distributing them across the groups at random. This helps to assemble GIs that are comparable in terms of their baseline characteristics, making attribution of any differences observed to the results of the intervention itself more reliable. To achieve this, in addition to rigorous inclusion criteria, it is imperative to employ techniques for randomization, blinding, and allocation to reduce selection biases and increase the experiment’s internal validity, maximizing the reliability and reproducibility of the results.^1,3,5^ Some examples of the main types of randomization and their characteristics are given in Chart 1 and 2 respectively.

**Chart 1:** Examples of methods for randomization of 32 participants to two intervention groups (A and B).

Type of randomization	Intervention	Sequence of allocation of the participants
**Simple**	A	1, 2, 4, 8, 9, 10, 15, 17, 21, 25, 26, 27, 28, 32
	B	3, 5, 6, 7, 11, 12, 13, 14, 16, 18, 19, 20, 22, 23, 24, 29, 30, 31
**In blocks**	A	1, 3, 6, 8, 9, 12, 14, 16, 17, 20, 21, 24, 26, 28, 29, 31
	B	2, 4, 5, 7, 10, 11, 13, 15, 18, 19, 22, 23, 25, 27, 30, 32
**Stratified**	A	Stratum X: 1, 3, 6, 8, 9, 12, 14, 16 Stratum Y: 17, 20, 21, 24, 26, 28, 29, 31
	B	Stratum X: 2, 4, 5, 7, 10, 11, 13, 15 Stratum Y: 18, 19, 22, 23, 25, 27, 30, 32
**Paired**	A	Var W: 1, 3, 6, 8, Var X: 9, 12, 14, 16 Var Y: 17, 20, 21, 24, Var Z: 26, 28, 29, 31
	B	Var W: 2, 4, 5, 7, Var X: 10, 11, 13, 15 Var Y: 18, 19, 22, 23, Var Z: 25, 27, 30, 32
**Adaptive by minimization**	A	1, 2, 4....dependent on the characteristics of initial recruits for pairing by characteristics.
	B	3, 5, 6
**Mendelian**	A	1, 2, 4....dependent on the characteristics of the initial recruits for genetic pairing.
	B	3, 5, 6

In its similarity to a simple lottery draw, simple randomization is technically best in terms of comprehensibility and feasibility. However, it can create IGs of disproportionate sizes and can cause imbalances in the proportion of covariates of interest when small samples are used (n < 100). In a study by Coelho et al.,^6^ 52 patients were randomized to receive elastic compression therapy for 7 days or to wear elastic stockings for 24 hours after phlebectomy. Demographic covariates were adequately homogeneous, despite a numerical imbalance between the IGs (n = 20 vs. N = 32 participants).

Block randomization guarantees that those allocated are distributed equally among the IGs, avoiding numerical disproportions between different interventions and enabling a certain degree of parallelism in allocations. However, for small samples there is still the risk of imbalance between relevant covariates between IGs. Additionally, in open trials, in which blocks are small, it is possible for researchers to anticipate which intervention will be drawn for the next participant to be allocated to a block, introducing selection bias.^7^ In a study by Garcia et al.,^8^ 20 participants were randomized in blocks to two IGs, to evaluate two training programs for patients with intermittent claudication. Although the IG sizes were balanced, the sample was too small to enable adequate homogenization of demographic covariates such as sex.

In order to minimize the risk that a covariate of relevance to the study outcome is imbalanced between the IGs and also enable stratified analyses of the results *a posteriori*, stratified block randomization should be used. In practice, this technique creates smaller strata with allocation blocks for individuals with or without the covariates of interest.^7^ An example would be stratification of patients with diabetes mellitus or smokers in trials of atherosclerosis treatments.

Randomized block allocation sequences, with or without stratification, can be generated online at sites such as GraphPad^9^and Research Randomizer.^10^

However, even with the stratification of blocks, the use of small samples can still cause inadequate homogenization of potential confounders such as age, sex, or ethnicity within the IGs. These disproportions should then be weighted in the analysis of the results using multivariate analyses. Paired randomization techniques can be used to create more strata or quotas for the inclusion of participants *a priori*. However, balancing the groups for pairing may make it difficult to recruit participants, delaying the study, since paired participants should ideally start their interventions in parallel.^7^

In order not to delay recruitment, adapted randomization by minimization aims to balance the IGs in terms of possible confounding factors, which occur dynamically during the recruitment phase. After initial simple randomization and allocation of some individuals, the baseline characteristics of the groups are analyzed and a calculation is performed to guarantee pairing and balanced stratification of subsequent participants recruited. This method requires software for continuous monitoring during the entire recruitment stage.^11^

In Mendelian randomization, genetic variants associated with the exposure of interest are used as pairing and stratification parameters, which requires prior knowledge of the genetic status of individuals eligible for the study within the population of interest.^12,13^

In general, simple randomization can be used if the sample size is greater than 100 participants per group. When smaller than this, block randomization guarantees better equilibrium of group sizes. However, if there is a need for *a posteriori* analysis by subsets (for example, disease severity, age group, sex/gender, body composition, prior treatments, comorbidities), randomization stratified by the variables of interest should be preferred.^14^ More elaborate methods, such as Mendelian, factorial, or cluster randomization, adaptive strategies, and minimization, should be supervised by an experienced statistics professional, with adequate computational support.^15-17^

Researchers should avail themselves of all available resources to guarantee a certain randomization for the allocation of participants to IGs. However, there are situations in which full randomization is not possible. In such cases, the method of non-randomized allocation that stands out is sequential (by convenience), in which participants are allocated based on a sequence defined at the time of recruitment. Quasi-randomized allocation can be used to minimize the burden of lack of randomization, in which a variable, non-random criterion is used to define the GI, such as study registration number (odd or even last number), date of birth, order of recruitment to the clinical trial, or day of the week. Moreover, in retrospective studies that compare interventions (for example, treatment cohorts) and other designs that use non-randomized allocation, conducting sensitivity analyses and multivariate adjustment for confounding variables is essential for ratification of the results.

The entire randomization process is intended to guide the allocation of participants to IGs in a homogeneous manner, according to the study characteristics.^18^ It is also important that the allocation process is protected from influence, whether intentional or otherwise, by members of the study research team, in order to minimize inclusion and detection biases. It is also of value to conduct allocation in a manner that precludes researchers from predicting the sequence of interventions, which could introduce bias through the selection of patients more or less favorable for the intervention.^19,20^

Allocation concealment is the term used to describe the randomization process in which the treatment allocated is unknown before the inclusion of the participant in the study.^21,22^ This can be achieved using opaque sealed envelopes numbered and organized in advance by someone external to the study team.^23,24^

Blinding, in turn, refers to masking the interventions from the individuals involved in the study after the allocation of participants. It can be applied to the participants (a blind study), to the participant and the investigator or rater of results (double-blind, rater-blind), or to everybody involved (triple-blind).^21^ However, it is not always possible to blind the entire research chain (participants, investigators, and raters) throughout the study and analysis of the results, especially not in surgical trials. In addition to selection bias, detection bias (of outcomes) and performance bias (of the interventions) should be considered in these cases.^25,26^ The research team must conscientiously ensure that at least one external rater is blinded to the IGs and that the outcomes are as objective as possible.

There are also exceptional situations that can make randomized allocation difficult since consent to randomization can be difficult to obtain if the patient has a preconceived idea about the interventions involved or when the study involves placebos.^26-29^ For ethical reasons, participants may have the right to agree to or refuse the IG allocation to which they are randomized. Trials with special designs (for example, Zelen design, double randomization, crossover trial) were developed to deal with such contingencies and take account of the reallocation of participants after initial randomization, but discussion of these designs is beyond the scope of this text.^30-32^

Finally, regardless of whether intervention studies benefit from techniques for randomization, blinding, and allocation that reduce selection and detection biases, it is still necessary to describe the methodology used in great detail, since this directly affects the internal validity of the results. Whether the methodological description is included on clinical trial registers or provided as supplementary material with the articles, it improves the reproducibility of the study and should be encouraged, even though only a small proportion of clinical trials present such details.^33^

**Chart 2:** Advantages and disadvantages of the main methods for randomization in clinical trials.

Randomization method	Advantages	Disadvantages
**Simple**	Easy to reproduce.	May cause imbalances between groups with small samples.
**In blocks**	Reduces imbalances between groups.	Allocation sequence can be inferred by researchers in open trials.
**Stratified in blocks**	Distributes possible predictive factors of the outcome equally across groups.	Generates very small groups when there are many strata, which compromises the power of the statistical analysis.
**Paired**	Reduces imbalances between groups.	Makes recruitment of patients more difficult, since it should be done simultaneously.
	Enables comparison of participants with the same predictive factors.	
**Adaptive by minimization**	Enables homogenization between groups to be performed as the study progresses.	Demands continuous monitoring with software.
**Mendelian**	Enables a constituent factor to be distributed homogeneously between groups.	May be influenced by other exposure factors with other variables that have not been considered (heterogeneous genetics, epigenetics, interaction between genes).

## References

[1] Altman DG, Bland JM (1999). Statistics notes. Treatment allocation in controlled trials: why randomise?. BMJ.

[2] Sacks H, Chalmers TC, Smith H (1982). Randomized versus historical controls for clinical trials. Am J Med.

[3] Roberts C, Torgerson D (1998). Understanding controlled trials: randomisation methods in controlled trials. BMJ.

[4] Miola AC, Miot HA (2021). P-value and effect-size in clinical and experimental studies. J Vasc Bras.

[5] Zhao W (2013). Selection bias, allocation concealment and randomization design in clinical trials. Contemp Clin Trials.

[6] Coelho F, Araujo WJB, Belczak S (2023). Influence of compression therapy following varicose vein surgery: a prospective randomized study. J Vasc Bras.

[7] Hulley SB, Cummings SR, Browner WS (2006). Designing clinical research..

[8] Garcia EL, Pereira AH, Menezes MG (2023). Effects of aerobic and combined training on pain-free walking distance and health-related quality of life in patients with peripheral artery disease: a randomized clinical trial. J Vasc Bras.

[9] GraphPad (2024). GraphPad.

[10] Research Randomizer (2024). Research Randomizer.

[11] Moher D, Hopewell S, Schulz KF (2010). CONSORT 2010 explanation and elaboration: updated guidelines for reporting parallel group randomised trials. BMJ.

[12] Ference BA, Holmes MV, Smith GD (2021). Using Mendelian randomization to improve the design of randomized trials. Cold Spring Harb Perspect Med.

[13] Davey Smith G, Hemani G (2014). Mendelian randomization: genetic anchors for causal inference in epidemiological studies. Hum Mol Genet.

[14] Kang M, Ragan BG, Park JH (2008). Issues in outcomes research: an overview of randomization techniques for clinical trials. J Athl Train.

[15] Pocock SJ, Simon R (1975). Sequential treatment assignment with balancing for prognostic factors in the controlled clinical trial. Biometrics.

[16] Taves DR (1974). Minimization: a new method of assigning patients to treatment and control groups. Clin Pharmacol Ther.

[17] Treasure T, MacRae KD (1998). Minimisation: the platinum standard for trials? Randomisation doesn’t guarantee similarity of groups; minimisation does. BMJ.

[18] Schulz KF, Grimes DA (2002). Generation of allocation sequences in randomised trials: chance, not choice. Lancet.

[19] Purssell E, Drey N, Chudleigh J, Creedon S, Gould DJ (2020). The Hawthorne effect on adherence to hand hygiene in patient care. J Hosp Infect.

[20] Chalmers TC, Celano P, Sacks HS, Smith H (1983). Bias in treatment assignment in controlled clinical trials. N Engl J Med.

[21] Forder PM, Gebski VJ, Keech AC (2005). Allocation concealment and blinding: when ignorance is bliss. Med J Aust.

[22] Berger VW, Do AC (2010). Allocation concealment continues to be misunderstood. J Clin Epidemiol.

[23] Pildal J, Chan AW, Hrobjartsson A, Forfang E, Altman DG, Gotzsche PC (2005). Comparison of descriptions of allocation concealment in trial protocols and the published reports: cohort study. BMJ.

[24] Schulz KF, Grimes DA (2002). Allocation concealment in randomised trials: defending against deciphering. Lancet.

[25] Kennedy CE, Fonner VA, Armstrong KA (2019). The Evidence Project risk of bias tool: assessing study rigor for both randomized and non-randomized intervention studies. Syst Rev.

[26] Deeks JJ, Dinnes J, D’Amico R (2003). Evaluating non-randomised intervention studies. Health Technol Assess.

[27] Popp L, Schneider S (2015). Attention placebo control in randomized controlled trials of psychosocial interventions: theory and practice. Trials.

[28] Linde K, Fassler M, Meissner K (2011). Placebo interventions, placebo effects and clinical practice. Philos Trans R Soc Lond B Biol Sci.

[29] Fregni F, Imamura M, Chien HF (2010). Challenges and recommendations for placebo controls in randomized trials in physical and rehabilitation medicine: a report of the international placebo symposium working group. Am J Phys Med Rehabil.

[30] Homer CS (2002). Using the Zelen design in randomized controlled trials: debates and controversies. J Adv Nurs.

[31] Simon GE, Shortreed SM, DeBar LL (2021). Zelen design clinical trials: why, when, and how. Trials.

[32] Torgerson DJ, Roland M (1998). Understanding controlled trials: What is Zelen’s design?. BMJ.

[33] Lai D, Wang D, McGillivray M, Baajour S, Raja AS, He S (2021). Assessing the quality of randomization methods in randomized control trials. Healthc (Amst).

