# Supplementary material for: Techniques for randomization and allocation for clinical trials
Source: J Vasc Bras. 2025 Jan 13;23:e20240046. doi: 10.1590/1677-5449.202400462 (PMC11758574; doi:10.1590/1677-5449.202400462)
Supplement: Translation

# Translation: Técnicas de randomização e alocação para estudos clínicos

**DOI:** 10.1590/1677-5449.202400461

**Authors:** Anna Carolina Miola, Ana Cláudia Cavalcante Espósito, Hélio Amante Miot

**Affiliations:** 1 Universidade Estadual Paulista – UNESP, Faculdade de Medicina de Botucatu, Botucatu, SP, Brasil.; 2 Universidade do Oeste Paulista – Unoeste, Presidente Prudente, SP, Brasil.

**Keywords:** Randomização, Ocultação de Alocação, Estudos de Intervenção, Redução de Viés, Validade Metodológica

## Abstract

Estudos de intervenção requerem que todos os participantes sejam provenientes da mesma população, com alocação aleatória aos grupos de intervenção (GI) para garantir comparabilidade. A randomização é fundamental para minimizar fatores de confusão, permitindo que diferenças nos resultados sejam atribuídas à intervenção. A randomização simples é eficaz para amostras grandes (>100 por grupo), mas amostras menores podem exigir randomização em blocos ou estratificada para equilibrar os tamanhos dos grupos e as covariáveis. Quando a randomização não é viável, métodos quasi-randomizados (como baseados em datas ou ordem de inclusão) podem ser utilizados, mas devem ser acompanhados de ajustes multivariados. Além disso, o cegamento e a ocultação da alocação aumentam a validade interna e a reprodutibilidade. A ocultação da alocação (ex.: envelopes lacrados) evita vieses durante a designação dos participantes, enquanto o cegamento reduz vieses de detecção e desempenho. Descrições metodológicas detalhadas em registros de ensaios clínicos e publicações aumentam a confiabilidade e a reprodutibilidade dos estudos, destacando a importância de um planejamento rigoroso e de relatórios transparentes em pesquisas de intervenção. Este artigo revisa os principais conceitos de randomização, cegamento e ocultação de alocação em estudos de intervenção.

A análise quantitativa de dados oriundos de estudos de intervenção demanda que a amostra seja proveniente da mesma população (em cada centro) e que a alocação nos grupos seja realizada de forma aleatória, isto é, sem interferência do pesquisador ou dos participantes. Isso garante que cada indivíduo tenha a mesma chance de ser designado para qualquer um dos grupos de intervenção (GIs)^1-4^ .

A importância da alocação randomizada (aleatória) reside na capacidade de homogeneizar fatores de confusão desconhecidos ou não mensuráveis, distribuindo-os aleatoriamente entre os grupos. Isso ajuda a criar GI comparáveis em termos de características basais, tornando mais confiável a atribuição de quaisquer diferenças nos resultados à intervenção propriamente dita. Nesse sentido, além de critérios rigorosos de inclusão, o uso de técnicas de randomização, cegamento e alocação é imperativo para reduzir vieses de seleção e aumentar a validade interna do experimento, maximizando a confiabilidade e reprodutibilidade dos resultados^1,3,5^ . Alguns exemplos e características dos principais tipos de randomização estão descritos nos Quadros 1 e 2.

**Quadro 1:** Exemplos de randomização de 32 participantes para dois grupos de intervenção (A e B).

Tipo de randomização	Intervenção	Sequência de alocação dos participantes
**Simples**	A	1, 2, 4, 8, 9, 10, 15, 17, 21, 25, 26, 27, 28, 32
	B	3, 5, 6, 7, 11, 12, 13, 14, 16, 18, 19, 20, 22, 23, 24, 29, 30, 31
**Em blocos**	A	1, 3, 6, 8, 9, 12, 14, 16, 17, 20, 21, 24, 26, 28, 29, 31
	B	2, 4, 5, 7, 10, 11, 13, 15, 18, 19, 22, 23, 25, 27, 30, 32
**Estratificada**	A	Estrato X: 1, 3, 6, 8, 9, 12, 14, 16 Estrato Y: 17, 20, 21, 24, 26, 28, 29, 31
	B	Estrato X: 2, 4, 5, 7, 10, 11, 13, 15 Estrato Y: 18, 19, 22, 23, 25, 27, 30, 32
**Pareada**	A	Var W: 1, 3, 6, 8, Var X: 9, 12, 14, 16 Var Y: 17, 20, 21, 24, Var Z: 26, 28, 29, 31
	B	Var W: 2, 4, 5, 7, Var X: 10, 11, 13, 15 Var Y: 18, 19, 22, 23, Var Z: 25, 27, 30, 32
**Adaptativa por minimização**	A	1, 2, 4....depende das características dos primeiros incluídos para pareamento de características.
	B	3, 5, 6
**Mendeliana**	A	1, 2, 4....depende das características dos primeiros incluídos para pareamento genético.
	B	3, 5, 6

Por se assemelhar a um simples sorteio, a randomização simples é, tecnicamente, melhor compreensível e implementável. No entanto, pode gerar GIs com tamanhos desproporcionais, além de provocar desequilíbrio na proporção de covariáveis de interesse quando se utilizam amostras pequenas (n < 100). No estudo de Coelho et al.^6^ , 52 pacientes foram randomizados para receber terapia compressiva elástica por 7 dias ou uso de meia elástica por 24 horas após flebectomia. Houve homogeneidade adequada das covariáveis demográficas, apesar do desequilíbrio numérico entre os GIs (n = 20 x 32 participantes).

A randomização em blocos garante que os sorteios sejam distribuídos igualmente dentro dos GIs, evitando desproporções numéricas entre as intervenções e permitindo certo paralelismo nas inclusões. Contudo, em amostras pequenas, também há o risco de desequilíbrio entre covariáveis relevantes dentro dos GIs. Ainda, em ensaios abertos, quando os blocos são pequenos, é possível que o pesquisador antecipe a intervenção sorteada para o próximo participante a ser incluído no bloco, causando viés de seleção^7^ . No estudo de Garcia et al.^8^ , 20 participantes foram randomizados em blocos para dois GIs, a fim de avaliar dois programas de treinamento para pacientes com claudicação intermitente. Apesar do equilíbrio nos tamanhos dos GIs, a amostra restrita não permitiu uma homogeneização adequada das covariáveis demográficas, como o sexo.

Para minimizar o risco de que uma covariável relevante para o desfecho do estudo esteja desequilibrada entre os GIs e permitir a realização de análise estratificada dos resultados *a posteriori*, deve-se utilizar a randomização estratificada em blocos, que, na prática, cria estratos menores com blocos de inclusão para indivíduos com ou sem as covariáveis de interesse^7^ . Um exemplo seria a estratificação de pacientes com diabetes melito ou tabagistas em ensaios terapêuticos de aterosclerose.

Sequências de alocação aleatorizadas em blocos, com ou sem estratificação, podem ser geradas *online* em *sites* como GraphPad^9^ e Research Randomizer^10^ .

Entretanto, mesmo com a estratificação dos blocos, a utilização de amostras pequenas ainda pode causar homogeneização inadequada de potenciais confundidores, como idade, sexo ou etnia, dentro dos GIs. Essas desproporções devem ser ponderadas na análise dos resultados por meio de ajustes multivariados. Técnicas de randomização pareada são utilizadas para criar, *a priori*, mais estratos ou cotas para a inclusão dos participantes. Entretanto, o balanceamento entre os grupos por pareamento pode dificultar o recrutamento de participantes e gerar atrasos no estudo, visto que os participantes pareados devem, preferencialmente, iniciar as intervenções de forma paralela^7^ .

Para não atrasar a inclusão, a randomização adaptativa por minimização visa equilibrar os GIs quanto a possíveis fatores confundidores, o que ocorre de forma dinâmica durante a fase de inclusão. Após uma randomização simples inicial e a alocação de alguns indivíduos, as características basais dos grupos são analisadas, e um novo cálculo é realizado para garantir o pareamento e a estratificação equilibrada dos novos participantes recrutados. Esse método exige o uso de *software* para monitoramento contínuo durante toda a fase de inclusão^11^ .

Na randomização mendeliana, variantes genéticas associadas à exposição de interesse são utilizadas como parâmetro de pareamento e estratificação, demandando um conhecimento prévio do estado genético dos indivíduos elegíveis para o estudo dentro de uma população de interesse^12,13^ .

De forma geral, a randomização simples pode ser empregada quando o tamanho amostral for maior que 100 participantes por grupo. Quando menor, a randomização em blocos garante um melhor equilíbrio no tamanho dos grupos. Entretanto, se houver a necessidade de análise posterior por subgrupos (por exemplo, gravidade da doença, faixa etária, gênero, composição corporal, tratamentos prévios, comorbidades), a randomização estratificada pelas variáveis de interesse deve ser preferida^14^ . Formas mais elaboradas, como a randomização mendeliana, fatorial, por conglomerados, estratégias adaptativas e de minimização, devem ser apoiadas por profissional estatístico experiente e suporte computacional adequado^15-17^ .

Os pesquisadores devem utilizar todos os recursos disponíveis para garantir a aleatoriedade na inclusão dos participantes nos GIs. Entretanto, há situações em que a randomização não é totalmente possível. Nesses casos, entre os métodos de alocação não randomizados, destaca-se o sequencial (por conveniência), no qual os participantes são alocados com base em uma ordem definida no momento da inclusão. Para minimizar o ônus da falta de randomização, podem-se utilizar alocações quasi-randomizadas, nas quais se usa um critério variável, mas não randomizado, de definição do GI, como número de registro no protocolo do estudo (final par ou ímpar), data de nascimento, ordem de recrutamento no ensaio clínico ou dia da semana. Ainda, em estudos retrospectivos que comparam intervenções (por exemplo, coortes terapêuticas) e outros desenhos que utilizam alocação não randomizada, a condução de análises de sensibilidade e o ajuste multivariado para as covariáveis de confusão são essenciais na ratificação dos resultados.

Todo o processo de randomização visa orientar a alocação dos participantes nos GIs de forma homogênea, de acordo com as características da pesquisa^18^ . É também importante que o processo de alocação seja protegido da influência, intencional ou não, dos membros pesquisadores do estudo, a fim de minimizar vieses de inclusão e de detecção. Ainda, é valioso que a alocação seja feita de forma que o pesquisador não possa predizer a sequência das intervenções, o que poderia enviesar a seleção de pacientes mais ou menos favoráveis à intervenção^19,20^ .

Ocultação de alocação é o termo usado para descrever o processo de randomização em que o tratamento a ser alocado não é conhecido antes da inclusão do participante no estudo^21,22^ . Isso pode ser realizado por meio de envelopes opacos e lacrados, enumerados e organizados previamente por um indivíduo externo à equipe do estudo^23,24^ .

O cegamento, por sua vez, refere-se ao mascaramento dos indivíduos envolvidos no estudo quanto às intervenções após a alocação dos participantes. Pode ser aplicado apenas ao participante (cego), ao participante em combinação com o investigador ou avaliador dos resultados (duplo-cego, avaliador-cego) ou a todos os envolvidos (triplo-cego)^21^ . Contudo, nem sempre é possível cegar toda a cadeia de pesquisa (participantes, investigadores e avaliadores) na execução do estudo e na análise dos resultados, especialmente em ensaios cirúrgicos. Além do viés de seleção, o viés de detecção (dos desfechos) e viés de performance (das intervenções) devem ser considerados nesses casos^25,26^ . A equipe de pesquisa deve se esmerar para que ao menos um avaliador externo seja cegado quanto aos GIs e que os desfechos avaliados sejam os mais objetivos possíveis.

Há, ainda, situações excepcionais que podem dificultar a alocação randomizada, já que o consentimento para a randomização pode ser difícil de ser obtido quando o paciente tem uma noção preconcebida sobre as intervenções envolvidas ou quando o estudo envolve placebo^26-29^ . Por questões éticas, o participante pode ter o direito de concordar ou não com a alocação no GI ao qual foi randomizado. Ensaios com desenhos especiais (por exemplo, desenho de Zelen, dupla randomização, *crossover*) foram desenvolvidos para contornar tais contingências e contemplam a realocação dos participantes após sua randomização inicial, mas sua discussão ultrapassa o escopo deste texto^30-32^ .

Por fim, embora estudos de intervenção sejam beneficiados por técnicas de randomização, cegamento e alocação que reduzam vieses de seleção e detecção, é necessária uma descrição minuciosa da metodologia utilizada, uma vez que isso afeta diretamente a validade interna dos resultados. A descrição metodológica, seja no registro dos ensaios clínicos ou na elaboração de materiais suplementares aos artigos, favorece a reprodutibilidade do estudo e deve ser incentivada, ainda que apenas uma fração pequena de ensaios clínicos apresente tal detalhamento^33^ .

**Quadro 2:** Vantagens e desvantagens dos principais métodos de randomização em estudos clínicos.

Método de randomização	Vantagens	Desvantagens
**Simples**	Fácil de ser reproduzida.	Pode haver desbalanço entre os grupos em amostras pequenas.
**Em blocos**	Reduz o desbalanço entre os grupos.	Sequência de alocação pode ser inferida pelo pesquisador em ensaios abertos.
**Estratificada em blocos**	Distribui um possível fator preditor do desfecho de forma igualitária entre os grupos.	No caso de muitos estratos, gera grupos muito pequenos, o que compromete o poder da análise estatística.
**Pareada**	Reduz o desbalanço entre os grupos.	Dificulta a inclusão de pacientes, que deve ser simultânea.
	Permite que participantes com os mesmos fatores preditivos sejam comparados.	
**Adaptativa por minimização**	Permite que a homogeneização entre os grupos ocorra simultaneamente ao desenvolvimento do estudo.	Exige monitorização contínua por *software*.
**Mendeliana**	Permite a distribuição de um fator constitucional de forma homogênea entre os grupos.	Pode sofrer influência de outros fatores de exposição com outras variáveis não consideradas (heterogeneidade genética, epigenética, interação entre genes).
